# Genetic characterization of H1N2 influenza a virus isolated from sick pigs in Southern China in 2010

**DOI:** 10.1186/1743-422X-8-469

**Published:** 2011-10-13

**Authors:** Wei Li Kong, Liang Zong Huang, Hai Tao Qi, Nan Cao, Liang Quan Zhang, Heng Wang, Shang Song Guan, Wen Bao Qi, Pei Rong Jiao, Ming Liao, Gui Hong Zhang

**Affiliations:** 1 College of Veterinary Medicine, South China Agricultural University, Guangzhou, China

## Abstract

In China H3N2 and H1N1 swine influenza viruses have been circulating for many years. In January 2010, before swine were infected with foot and mouth disease in Guangdong, some pigs have shown flu-like symptoms: cough, sneeze, runny nose and fever. We collected the nasopharyngeal swab of all sick pigs as much as possible. One subtype H1N2 influenza viruses were isolated from the pig population. The complete genome of one isolate, designated A/swine/Guangdong/1/2010(H1N2), was sequenced and compared with sequences available in GenBank. The nucleotide sequences of all eight viral RNA segments were determined, and then phylogenetic analysis was performed using the neighbor-joining method. HA, NP, M and NS were shown to be closely to swine origin. PB2 and PA were close to avian origin, but NA and PB1were close to human origin. It is a result of a multiple reassortment event. In conclusion, our finding provides further evidence about the interspecies transmission of avian influenza viruses to pigs and emphasizes the importance of reinforcing swine influenza virus (SIV) surveillance, especially before the emergence of highly pathogenic FMDs in pigs in Guangdong.

## Findings

Influenza viruses are members of family Orthomyxoviridae and have segmented, Negative-sense RNA genomes. Swine influenza virus (SIV) belongs to Influenza A Viruses. SIV causes respiratory diseases in pigs and has been disseminated all over the world [1]. At present, there were three main influenza viruses circulating in pigs in the world including H1N1, H1N2 and H3N2. In addition some other unusual subtypes of swine influenza were also reported includingH1N7, H3N1, H4N6, H5N1, H5N2, H6N6 and H9N2 [2-7].

The first SIV H1N2 was reported in Japan from 1978 to 1992 [8,9]. From then on, H1N2 was show up in different pigs of different countries, including France from 1987 to 1988 [10], and in the United Kingdom in 1994 [11], the United States in 1999 [12], Germany in 2000 [13], Korea in 2003 and thereafter [14]. Recently it first shows up in Swedish herd [15]. During an influenza virus surveillance programme in Guangdong pigs, we isolated an H1N2 virus from clinically ill pigs, which was genetically characterized as a result of reassortment events between a human H3N2 strain, Classical SIV strain and North America avian-like SIV lineage strain.

In January of 2010, some pigs have a severe outbreak of influenza-like disease occurred in an intensive pig farm of Guangdong province. Many pigs have similar clinical symptoms: cough, sneeze, runny nose. These clinical symptoms last for 3-8 days then some pigs have sick of foot and mouth disease (FMD). Because Swine influenza (SI) was immunosuppressive disease which frequently predisposesed to highly fatal secondary infections. Maybe the SI lowers pig's immunity to common illnesses, some pigs will get FMD. From now on, the FMD caused rampant epidemic diseases in pig population of Guangdong [16].

Initial isolations of the viruses were performed in 10-day-old specific pathogen free (SPF) embryonated chicken eggs through the allantoic route, incubated at 35°C for 72 h. Embryonic death was monitored every 12 h, and then allantoic fluid were harvested under aseptic conditions and stored at -70°C for reserved. Subtype identification were conducted through RT-PCR and through standard hemagglutination inhibition and neuraminidase inhibition assays. One influenza virus was isolated and named: A/swine/Guangdong/1/2010(H1N2).

The virus RNA was extracted from allantoic fluid by using TRIzol reagents (Invitrogen). RT-PCR was performed as a one-step reaction with the TAKARA OneStep RT-PCR Kit, according to manufacturer's protocol. The primer of reverse transcription used 12 bp (5-AGC AAA AGC AGG-3). cDNAs were synthesized at 37°C for 1 h using M-MLV reverse-transcription system (Promega). Full-length PCR amplification of eight RNA segments was performed with a set of primer. The genome of this H1N2 SIV was sequenced fully as described previously, with the GenBank accession numbers HQ85339 to HQ853346.

Phylogenetic analysis of A/swine/Guangdong/1/2010(H1N2) was carried out by analyzing the data obtained here with those of other sequences of influenza viruses from GenBank database. A neighbor-joining nucleic acid tree was constructed in MEGA 4.0 using the Kimura 2-parameter model with 1, 000 bootstrap replicates. Clustal W of Lasergene was used for multiple alignments. In this study, the nucleotide sequences used for the phylogenetic analysis are as follows: PB2 (nt 100-2307), PB1 (nt 468-2297), PA (nt 479-2150), HA (nt 65-1689), NP (nt 45-1563), NA (nt 35-1422), M (nt 25-996), NS (nt 41-852). Table 1 lists the reference viruses with the highest level of sequence identity to A/swine/Guangdong/1/2010 for each gene segment.

**Table 1:** Sequence homology of each gene from A/swine/Guangdong/1/2010 (H1N2) and reference virus sequences available in GenBank.

Gene	Region	Virus with the highest degree of homology	Nucleotide sequence Identity (%)	Influenza virus lineage
HA	65-1689	A/swine/Beijing/47/1991(H1N1)[U46783]	94.6	Swine
NA	35-1422	A/Turkey/MO/24093/99(H1N2)[AY038015]	97.4	Human
PB2	100-2307	A/swine/HongKong/NS623/2002(H1N2)[GQ229369]	98.1	Avian
PB1	468-2297	A/turkey/Ontario/31232/2005(H3N2)[DQ469996]	99.8	Human
PA	479-2150	A/swine/HongKong/NS623/2002(H1N2)[GQ229368]	97.5	Avian
NP	45-1563	A/swine/Hong Kong/915/2004(H1N2) [GQ229270]	98.0	Swine
M	25-996	A/swine/HongKong/NS623/2002(H1N2)[GQ229367]	98.9	Swine
NS	41-852	A/swine/Hong Kong/1110/2006(H1N2) [GQ229371]	97.6	Swine

**Figure 1:**
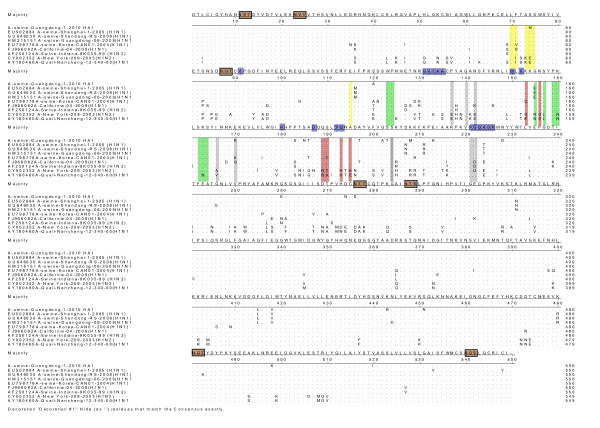
**Molecular of the HA gene of the H1N2 SI virus and reference strains**. Green horizontal box is potential glycosylation sites;undertint horizontal box is receptor binding sites; antigen site Sa is reseda shadow, Sbis red shadow, Cais pink shadow, Cbis yellow shadow.

The HA deduced amino acid sequences of the A/swine/Guangdong/1/2010 was analyzed in this study. There were eight potential glycosylation sites in HA, including six in HA1 and two in HA2. Its cleavage site is (PSIQSRGLFGAI), which is not same with the HPAI (RXR/KRGLF) of the 1918 influenza virus which have strong pathogenic effect on the human (Figure 1).

**Figure 2:**
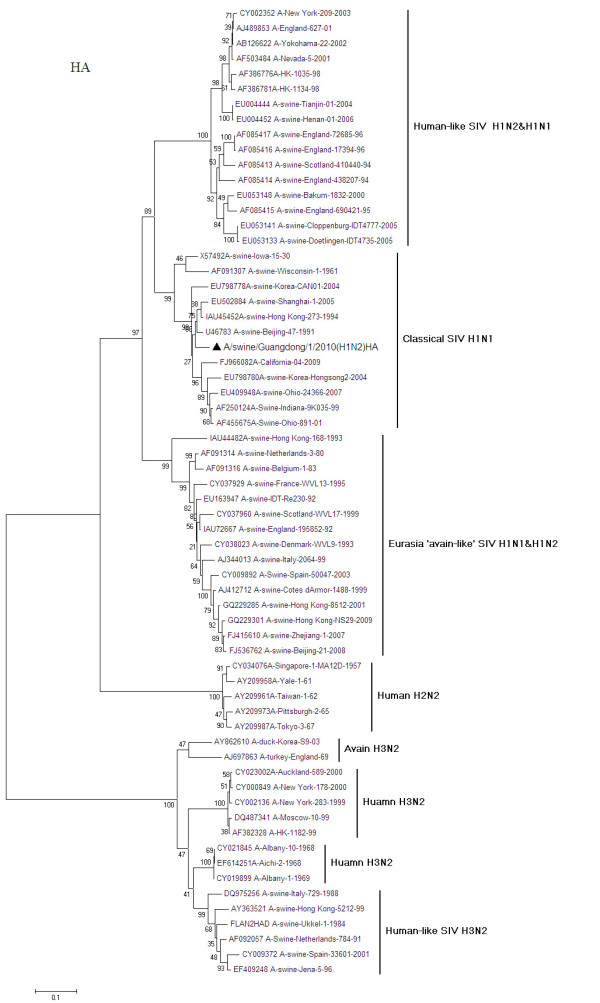
**Phylogenetic relationships of the HA gene of A/swine/Guangdong/1/2010 (H1N2) compared to genetically related influenza viruses**. Horizontal distances are proportional to the minimum number of nucleotide differences required to join nodes and sequences. Vertical distances are for spacing branches and labels. The phylogenetic trees were generated by using the neighbor-joining alogrithm. The percentage of replicate trees in which the associated taxa clustered together in the bootstrap test (1000 replicates) is shown next to the branches.

Phylogenetic analysis revealed that the HA gene of A/swine/Guangdong/1/2010 is of classical swine lineage resides in the same clade with pandemic (H1N1) 2009A/California/04/2009, along with A/swine/Beijing/47/1991(H1N1) and A/Swine/Indiana/9K035/99(H1N2) (Figure 2). Sequence homology with A/swine/Beijing/47/1991(H1N1) and A/swine/Hong Kong/273/1994(H1N1) was respectively found to be 94.6% and 93.9%. It still has a high homology with A/Swine/Indiana/9K035/99(H1N2) with 92.1%, which is the first reported triple reassortment swine influenza virus H1N2 in United States [12]. A/swine/Cloppenburg/IDT4777/2005(H1N2) is a novel SIV H1N2 isolated from Germany, whose HA gene originated from a human epidemic strain, is clearly distinct in genetic origin compared with those classical SIVs. Therefore, it is obviously that the HA gene of A/swine/Guangdong/1/2010 is a descendent of classical SIV and originated from the HA gene of neither avian origin nor human origin.

**Figure 3:**
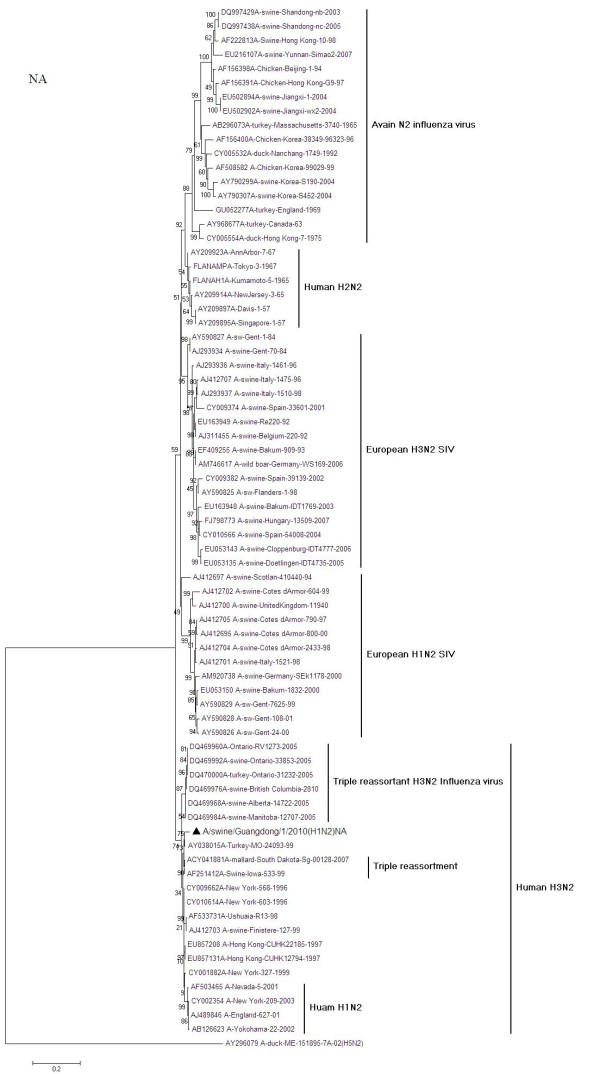
**Phylogenetic relationships of the NA gene of A/swine/Guangdong/1/2010 (H1N2) compared to genetically related influenza viruses**. Horizontal distances are proportional to the minimum number of nucleotide differences required to join nodes and sequences. Vertical distances are for spacing branches and labels. The phylogenetic trees were generated by using the neighbor-joining alogrithm. The percentage of replicate trees in which the associated taxa clustered together in the bootstrap test (1000 replicates) is shown next to the branches.

The phylogeny of the NA genes (Figure 3) also demonstrated that A/swine/Guangdong/1/2010 is genetically related to A/New York/568/1996(H3N2) and that the NA genes of triple reassortment H3N2(TR-H3N2) North American human [A/Ontario/RV1273/2005] and swine [A/swine/Ontario/33853/2005] and avian [A/mallard/South Dakota/Sg-00128/2007] influenza virus have established themselves as a single clade [17,18]. This clade shares a common ancestor with the human H3N2 viruses isolated during the 1990s in New York [A/New York/568/1996(H3N2)]. Comparison of partial sequences of the NA genes between TR-H3N2 and A/Turkey/MO/24093/99(H1N2) [19] showed high value of identities (94.0%-97.2% and 97.4%, respectively), which suggested close relationship between these viruses. Sequence homology (bp) with A/New York/568/1996 was identified as 96.5%. When compared with European H3N2 SIV and Avian-like SIV, a low degree of similarity is respectively observed as 85.3%-87.8% and 81.8%-84.9%.

Phylogenetic trees of the nucleotide sequences of NP, M and NS were involved in the classical SIV lineage. Different subtype triple reassortment influenza viruses still stand with them in the same clade. PB2 and PA was likely originated from North America avian-like SIV lineage. PB1 was close to human H3N2 lineage as the NA gene. (By way of example, the phylograms for the PB1, PB2, PA, NP, M and NS genes of the viruses featured in the present study are available in the additional files 1, 2, 3, 4, 5 and 6).

The PB1-F2 protein of influenza A virus encoded by an alternative reading frame in the PB1 polymerase gene, contains various lengths, amino acid sequences, cellular localizations and functions, which in terms displays strain-specific pathogenicity. The PB1-F2 of A/swine/Guangdong/1/2010 expressed a truncated protein including 57 amino acid like most human H1N1 viruses, which has a low homology with A/New York/568/1996 and other triple reassortment influenza virus (86.2%). It is different from A/Swine/Indiana/9K035/99 which coded full-length PB1-F2. The truncated protein may change the levels of expression and cellular localizations [20]. The NS1 protein of this virus contains 219 amino acid, it loss the PL motifs (PDZ domain ligand, PL), which located in 227-230aa. This feature is the same with recent classical SIV H1N1 [21].

In China, classical swine influenza virus has been existing since 1996. In the previous epidemiological study, H3N2 infected pigs in this area. The H1N2 was reported in 2004, which is a human-swine reassortment virus [22]. In this study we find the multiple reassortment viruses, it confirmed the swine as a mixing vessel in transmission. This deserves to be paid attention. Especially swine population was seized with FMD after the SIV infected pigs in this area. The 2009 H1N1 pandemic virus is a swine-origin reassortant: HA, NP and NS from classical swine (North American) lineage; PB2 and PA from avian (North American) lineage; PB1 from human seasonal H3N2; and NA and M from Eurasian swine lineage [23]. Southern China is designated as a putative influenza epicenter [24]. Both 1957 and 1968 pandemic influenza emerged from this area. The precursors of currently still ongoing H5N1 HPAIVs were identified also in Southern China. Recently H6 AIVs have been identified in this area [25]. The current situation, therefore, presents continued risk for further reassortment of swine influenza virus in pig populations and continuous monitoring of this virus in swine population will be needed.

## Nucleotide sequence accession numbers

Nucleotide sequences from the A/swine/Guangdong/1/2010 (H1N2) isolate have been submitted to GenBank with accession numbers HQ85339 to HQ853346.

## Competing interests

The authors declare that they have no competing interests.

## Authors' contributions

WLK carried out PCR and sequencing reactions and optimized protocols and performed sequence analyses, alignments, phylogenies, interpretation of data, carried out identification of viruses and wrote the manuscript. NC and HTQ revised the manuscript. LZH obtained the clinical samples, organized sample processing. All authors read and approved the final manuscript.

## Additional file

Phylogenetic trees for H1N2 swine influenza virus isolated from southern China: (1) PB2; (2) PB1; (3) PA; (4) NP; (5) MP; and (6) NS

## Supplementary Material

Additional file 1 **Phylogenetic relationships of the PB2 gene of A/swine/Guangdong/1/2010 (H1N2) compared to genetically related influenza viruses**. Click here for file

Additional file 2 **Phylogenetic relationships of the PB1 gene of A/swine/Guangdong/1/2010 (H1N2) compared to genetically related influenza viruses**. Click here for file

Additional file 3 **Phylogenetic relationships of the PA gene of A/swine/Guangdong/1/2010 (H1N2) compared to genetically related influenza viruses**. Click here for file

Additional file 4 **Phylogenetic relationships of the NP gene of A/swine/Guangdong/1/2010 (H1N2) compared to genetically related influenza viruses**. Click here for file

Additional file 5 **Phylogenetic relationships of the MP gene of A/swine/Guangdong/1/2010 (H1N2) compared to genetically related influenza viruses**. Click here for file

Additional file 6 **Phylogenetic relationships of the NS gene of A/swine/Guangdong/1/2010 (H1N2) compared to genetically related influenza viruses**. Click here for file
