# Supplementary figures and images for: Genetic characterization of H1N2 influenza a virus isolated from sick pigs in Southern China in 2010
Source: Virol J. 2011 Oct 13;8:469. doi: 10.1186/1743-422X-8-469 (PMC3221723; doi:10.1186/1743-422X-8-469)

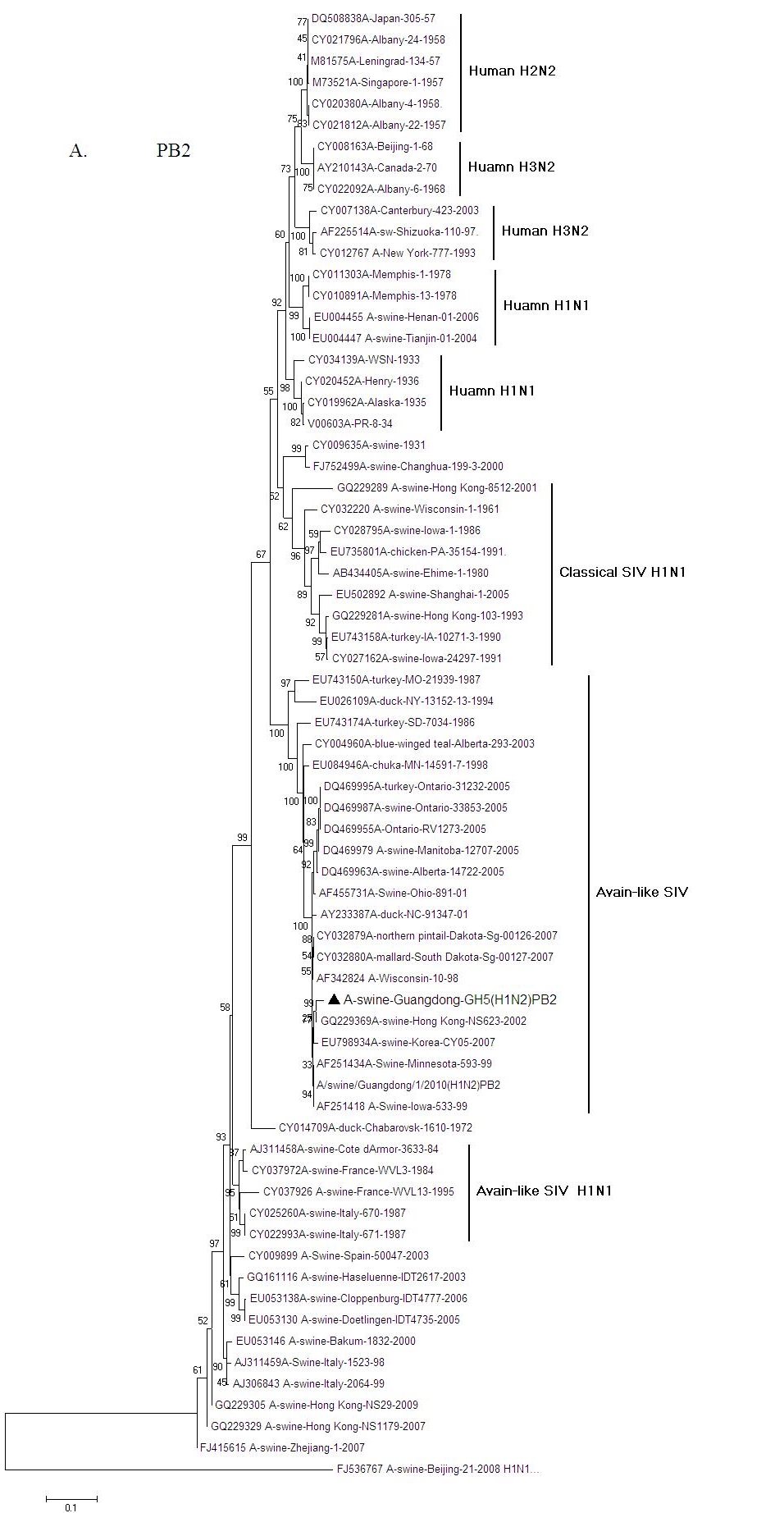

Supplement: Additional file 1 — Phylogenetic relationships of the PB2 gene of A/swine/Guangdong/1/2010 (H1N2) compared to genetically related influenza viruses. [file 1743-422X-8-469-S1.JPEG]

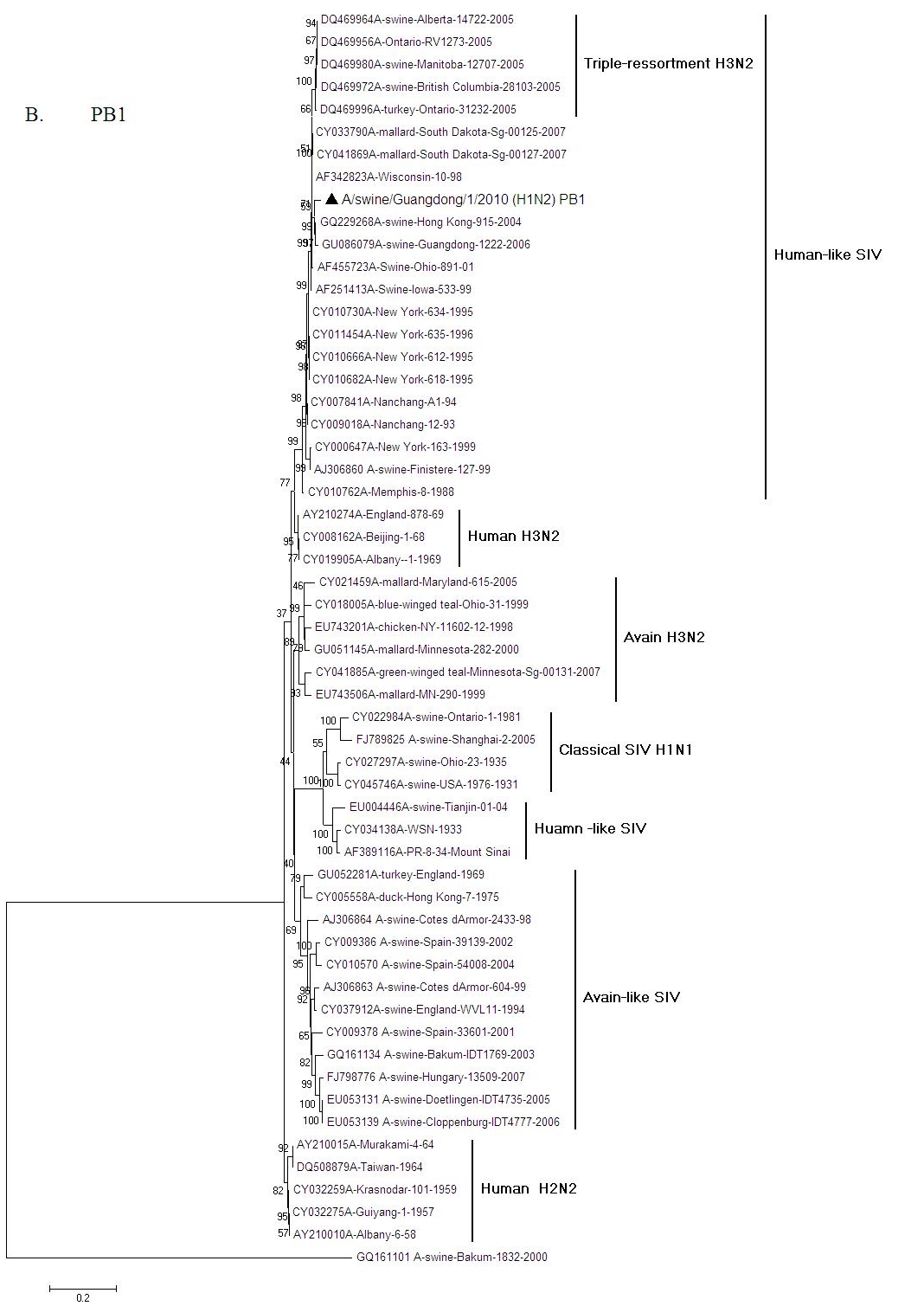

Supplement: Additional file 2 — Phylogenetic relationships of the PB1 gene of A/swine/Guangdong/1/2010 (H1N2) compared to genetically related influenza viruses. [file 1743-422X-8-469-S2.JPEG]

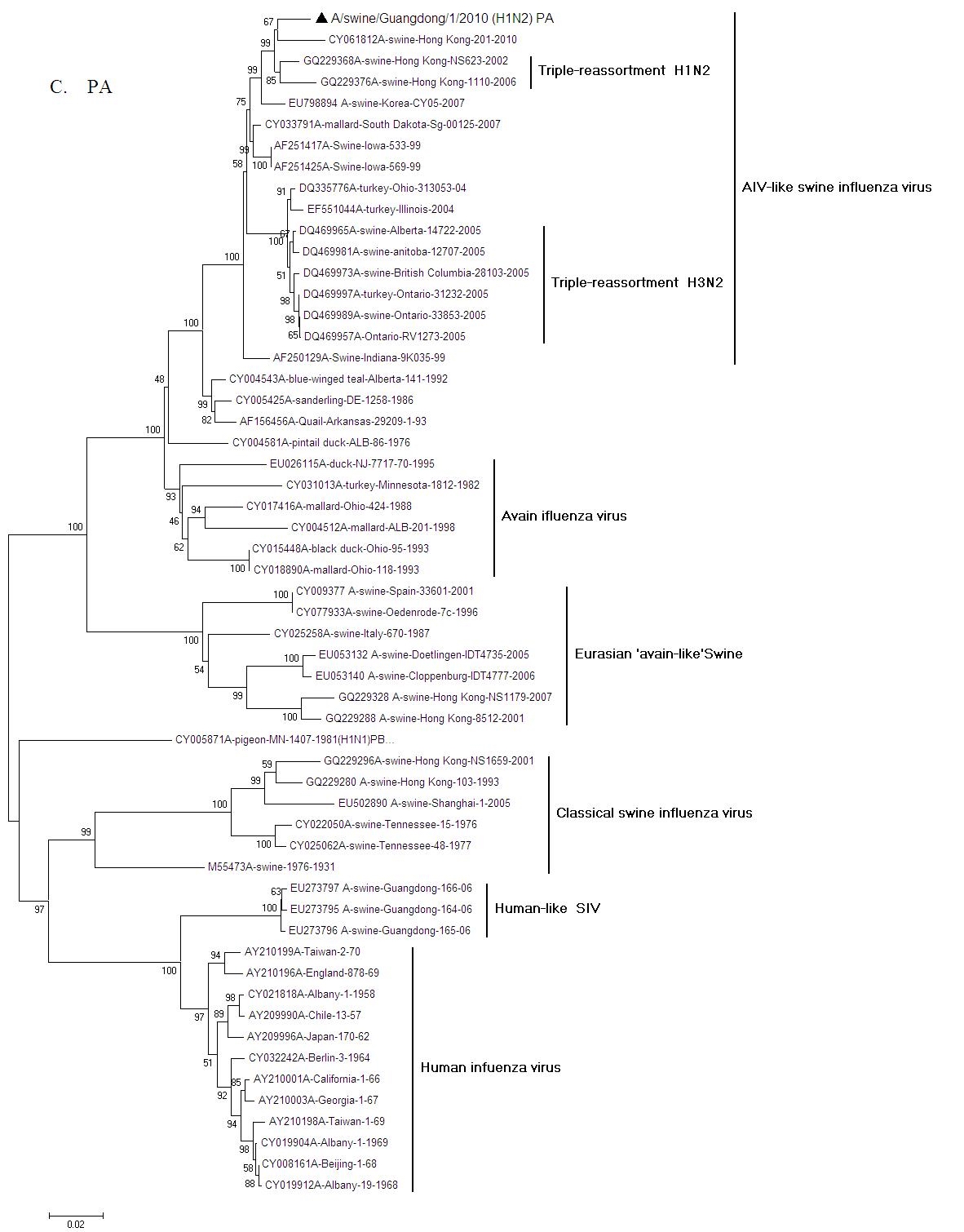

Supplement: Additional file 3 — Phylogenetic relationships of the PA gene of A/swine/Guangdong/1/2010 (H1N2) compared to genetically related influenza viruses. [file 1743-422X-8-469-S3.JPEG]

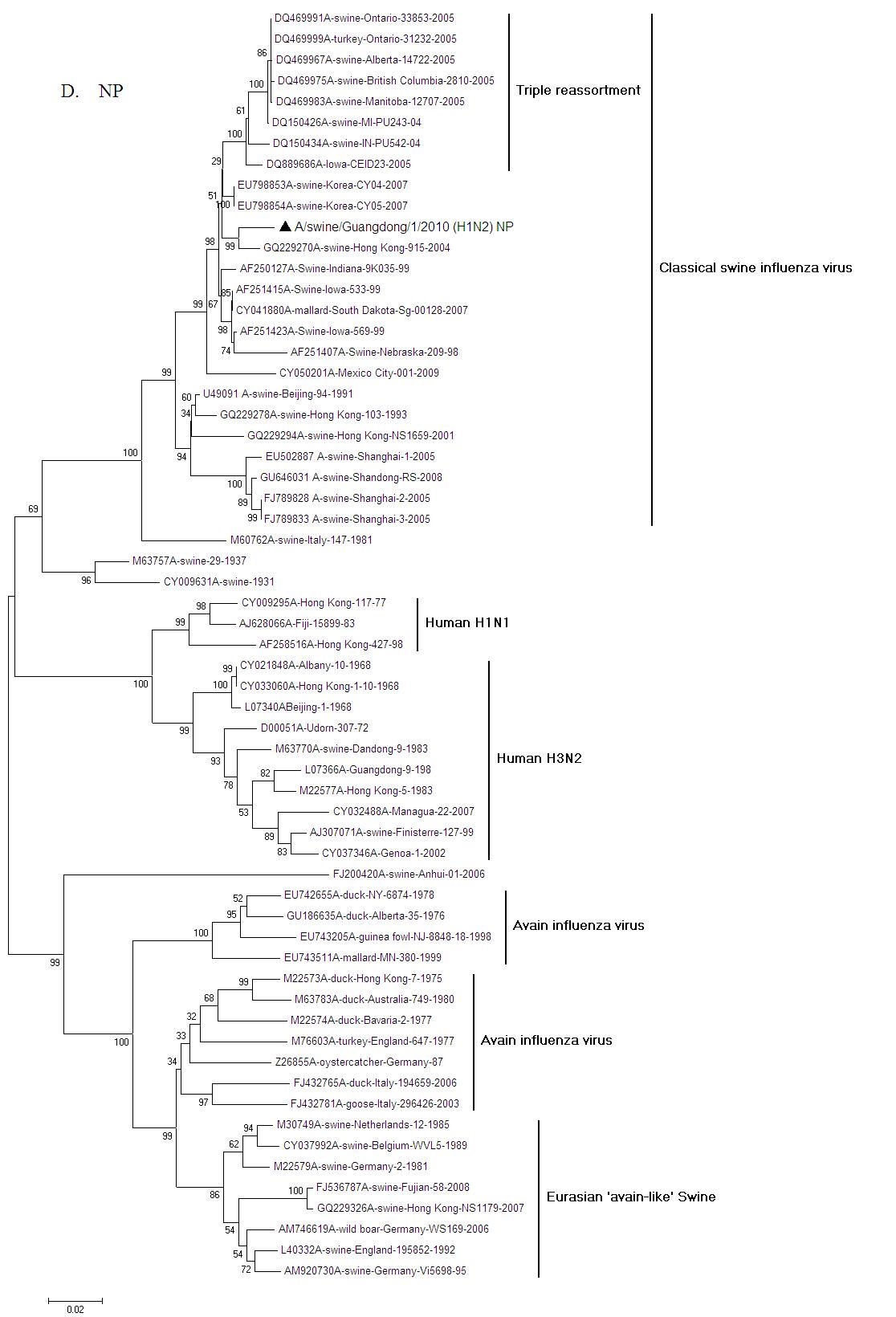

Supplement: Additional file 4 — Phylogenetic relationships of the NP gene of A/swine/Guangdong/1/2010 (H1N2) compared to genetically related influenza viruses. [file 1743-422X-8-469-S4.JPEG]

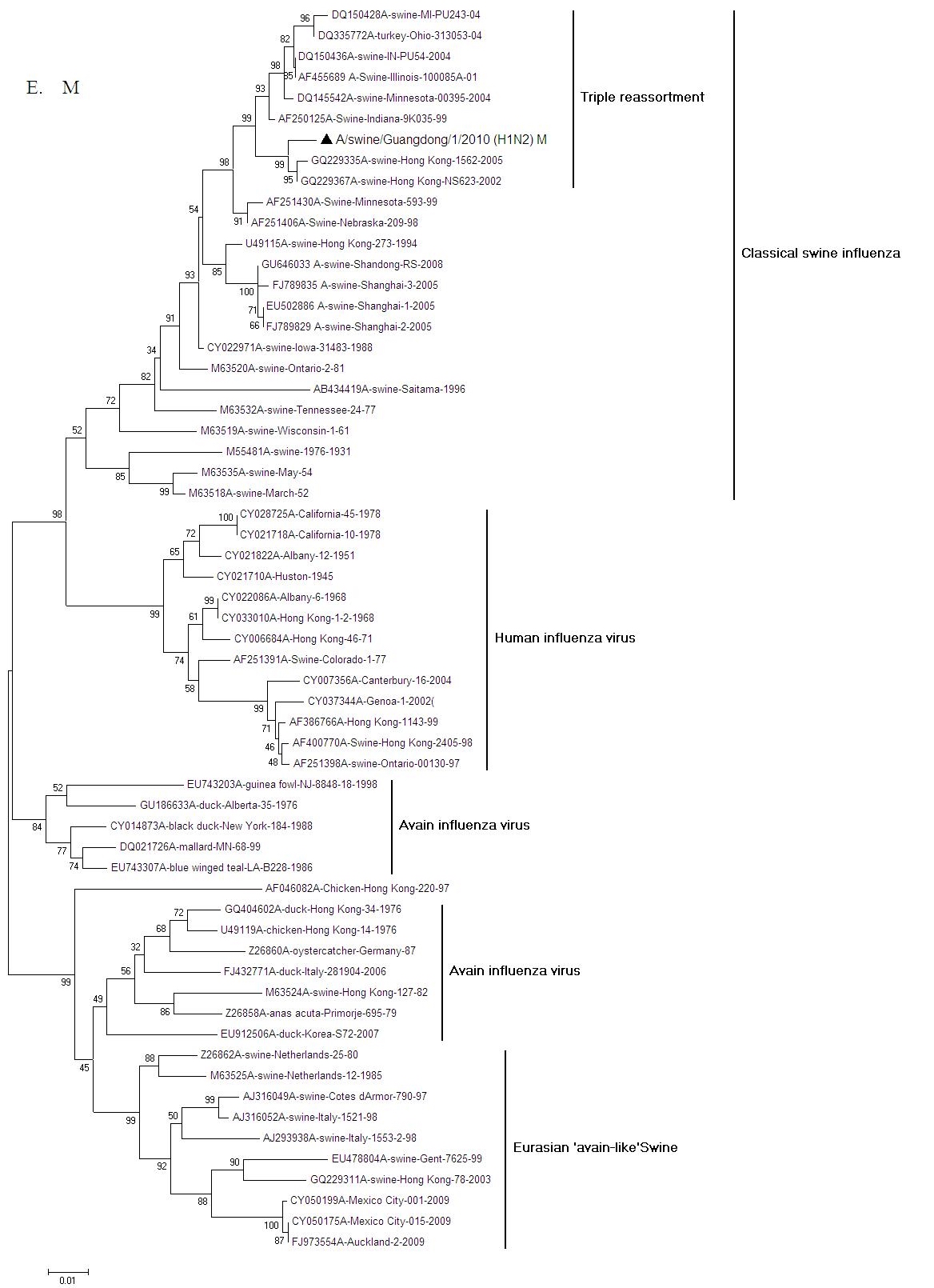

Supplement: Additional file 5 — Phylogenetic relationships of the MP gene of A/swine/Guangdong/1/2010 (H1N2) compared to genetically related influenza viruses. [file 1743-422X-8-469-S5.JPEG]

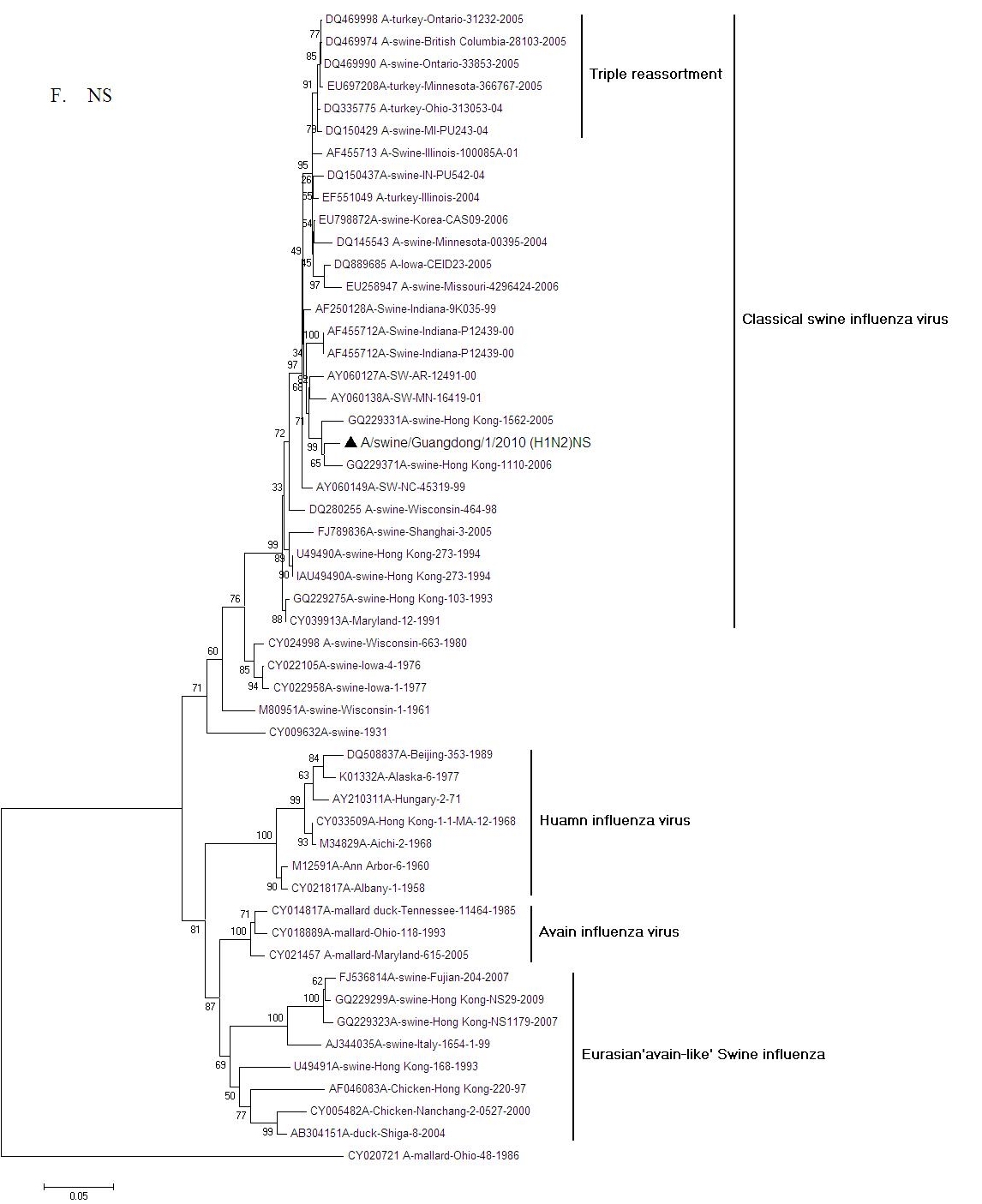

Supplement: Additional file 6 — Phylogenetic relationships of the NS gene of A/swine/Guangdong/1/2010 (H1N2) compared to genetically related influenza viruses. [file 1743-422X-8-469-S6.JPEG]
